# Corrosion-Resistive ZrO_2_ Barrier Films on Selected Zn-Based Alloys

**DOI:** 10.3390/ma16247673

**Published:** 2023-12-15

**Authors:** Irina Stambolova, Daniela Stoyanova, Maria Shipochka, Nelly Boshkova, Silviya Simeonova, Nikolay Grozev, Georgi Avdeev, Ognian Dimitrov, Nikolai Boshkov

**Affiliations:** 1 Institute of General and Inorganic Chemistry, Bulgarian Academy of Sciences, “Acad. G. Bonchev” St. Bl. 11, 1113 Sofia, Bulgaria; dsto@svr.igic.bas.bg (D.S.); shipochka@svr.igic.bas.bg (M.S.); 2 Institute of Physical Chemistry “R. Kaishev”, Bulgarian Academy of Sciences, 1113 Sofia, Bulgaria; nelly.boshkova@ipc.bas.bg (N.B.); g_avdeev@ipc.bas.bg (G.A.); nboshkov@ipc.bas.bg (N.B.); 3 Faculty of Chemistry and Pharmacy, Sofia University, 1164 Sofia, Bulgaria; fhsss@chem.uni-sofia.bg (S.S.); fhng@chem.uni-sofia.bg (N.G.); 4 Institute of Electrochemistry and Energy Systems, Bulgarian Academy of Sciences, “Acad. G. Bonchev” St. Bl. 10, 1113 Sofia, Bulgaria; ognian.dimitrov@iees.bas.bg

**Keywords:** zirconium oxide films, zinc alloy coatings, surface morphology, corrosion resistance, structure

## Abstract

This work presents the enhanced corrosion resistance of newly developed two-layer composite coatings deposited on low-carbon steel: electrodeposited zinc alloy coatings (Zn–Ni with 10 wt.% Ni (ZN) or Zn–Co with 3 wt.% Co (ZC), respectively) and a top ZrO_2_ sol–gel layer. Surface morphology peculiarities and anti-corrosion characteristics were examined using scanning electron microscopy (SEM), energy dispersive spectroscopy (EDX), atomic force microscopy (AFM), water contact angle (WCA) measurements, X-ray diffraction (XRD), X-ray photoelectron spectroscopy (XPS) analyses, potentiodynamic polarization (PDP) curves, corrosion potential (Ecorr), polarization resistance (Rp) measurements (for a prolonged period of 25 days) and open-circuit potential (OCP). The results were compared with the corrosion peculiarities of usual zinc coating. The zirconia top coatings in both systems were amorphous and dense, possessing hydrophobic nature. The experimental data revealed an increased corrosion resistance and protective ability of the ZC system in comparison to that of ZN due to its smooth, homogeneous surface and the presence of poorly crystallized oxides (ZnO and Co_3_O_4_), both later playing the role of a barrier for corrosive agents.

## 1. Introduction

Zirconium oxide is an important ceramic material used in many branches of industry: optics, biomedicine, etc. It has a wide band gap and a very high thermal expansion coefficient, compatible with many bulk metals. ZrO_2_ also exhibits high chemical stability, wear resistance and hardness, which are the prerequisites for effective corrosion protection [1,2]. Zirconia-based coatings can be prepared using various physical and chemical techniques, such as physical vapor deposition (PVD) [3], magnetron sputtering [4], chemical vapor deposition (CVD) [5], plasma spray [6], hydrothermal process [7] and sol–gel process [8].

Chemical deposition techniques are widely used due to the low cost of the equipment, low process temperature and the possibility of obtaining films on large areas for the easy control of the films’ stoichiometry, the degree of crystallization, etc. 

In the current literature, the data devoted to the investigation of anti-corrosion properties of chemically deposited ZrO_2_ on galvanized steel are very scarce. Recently, only a handful of authors have successfully applied the spray method for the deposition of ZrO_2_ coatings on some hot-dip galvanized low-carbon steel types (the latter containing Al between 5 and 55 wt.%) with the thickness of the hot-dip (zinc) coating within the range of 13–30 µm. Zirconium acetylacetonate–water or ethanol–water solutions were used as the precursor. Samples with deposited ZrO_2_ layers were tested in 0.5 M NaCl solution, and they showed good anti-corrosion performance compared to pure galvanized steel substrates [9,10,11,12].

The sol–gel method is a low-temperature method, and it is financially beneficial, enabling the possibility of obtaining a wide spectrum of oxide coatings. It also offers good adhesion to metallic surfaces, which is a very important advantage in the preparation of corrosion-resistive coatings [13]. The sol–gel method is an interesting, feasible alternative for the preparation of corrosion-protective layers on metallic substrates with various shapes and sizes, which make them suitable for industrial applications. Nevertheless, in the available literature to date, the data regarding investigations devoted to systems composed of sol–gel ZrO_2_ coatings deposited on steel and galvanized steel are not observable.

The corrosion of metals is a very common worldwide problem, arising from their interaction with the environment and leading to great financial, material, environmental and sometimes also human losses [14]. It is well known that iron corrodes, forming porous layers of corrosion products (red rust) and thus enabling a relatively easy access of the corrosive agents deep inside the bulk materials. For this reason, the corrosion resistance of metallic materials needs to be improved. As is well known, galvanic zinc and zinc alloy plating seems to be a widespread method for protection of low-carbon steels. The anti-corrosive resistance of zinc concerning steel (Fe) is due to the potential difference between these metals, leading to anodic dissolution of Zn. The latter plays a sacrificial role and dissolves first in the case of corrosion attack, leading to the appearance of corrosion products with low solubility value and composition, which depends on the composition of the corrosive medium; for example, zinc hydroxide chloride appears in the case of a 5% NaCl medium. These corrosion products create an additional protective layer with a barrier characteristic, inhibiting the penetration of an aggressive medium deep inside [15,16,17]. One possible disadvantage of this method could be the insufficient protection of the steel substrate only from zinc corrosion products in very aggressive media. In order to further protect zinc-coated steel against corrosion, surface modification is generally adopted. The good corrosion resistance of galvanized steel could be improved by alloying Zn with selected metals, such as Co, Ni, Mn, Fe, etc., which generally leads to higher polarization resistance values in chlorine-containing medium compared to a case where ordinary Zn is used as a substrate [16]. Another approach for enhanced corrosion protection is the application of some inhibitors [18].

In the present paper, we aim to prolong the lifetime of low-carbon steel in chlorine-containing media by means of newly developed systems composed of selected Zn alloys (Zn modified with 10 wt.% Ni or 3 wt.% Co) applied as sub-layers, the latter being covered by sol–gel ZrO_2_ films. We also test the behavior of these new systems for a prolonged period of time, aiming to check their suitability for some potential industrial applications.

## 2. Materials and Methods

### 2.1. Deposition of the Samples

Low-carbon steel plates (Metalsnab, Sofia, Bulgaria) with dimensions of 30 mm × 10 mm × 1 mm and the following elemental composition balance (wt.%) (C 0.05–0.12; S ≤ 0.04; P ≤ 0.35; Mn 0.25–0.5; Cr ≤ 0.1; Si ≤ 0.03; Ni ≤ 0.3; Cu ≤ 0.3; As ≤ 0.08; Fe) were taken as substrates. Two kinds of systems were realized and tested:

- System ZN: Zn–Ni (10 wt.%)—sub-layer; ZrO_2_—top layer;
- System ZC: Zn–Co (3 wt.%)—sub-layer; ZrO_2_—top layer.

System ZN was prepared using the following procedures: First, the alloy coating Zn–Ni (10 wt.%) was electrodeposited in an experimental cell via circulation from an electrolyte having a composition NiSO_4_·7H_2_O:NiCl_2_·6H_2_O:ZnCl_2_:β-alanine—100:100:30:10 (g/L). All the constituents were purchased from Valerus Ltd., Sofia, Bulgaria. The pH value was 4. The electrodeposition process was held using a cathodic current density of 2 A/dm^2^, a temperature of 40 °C and applying non-soluble Ti-Pt networks as anodes [16]. Afterward, the zirconia-based top films were prepared using zirconium butoxide—Zr(OC_4_H_9_)_4_ (Sigma Aldrich, St. Louis, MO, USA; CAS No.: 1071-76-7, 80 wt.% in 1-butanol, molecular weight: 383.68). The Zr precursor was diluted by adding isopropanol and some amounts of acetyl acetone (Sigma Aldrich, CAS No.: 123-54-6, ≥99%, molecular weight: 100.12) and acetic acid (Sigma Aldrich, CAS No.: 64-19-7, purity 99.8%, molecular weight: 60.05) as complexating agents in order to obtain 0.2 M solution. The next step was to add nitric acid to prevent hydrolysis (Sigma Aldrich, CAS No.: 7697-37-2, purity 99.8%, molecular weight: 63.01) and polyethylene glycol (PEG 400; Sigma Aldrich, CAS No.: 25322-68-3). The final solution was stirred for 2 h until the appearance of a yellowish transparent color. The low-carbon steel samples, previously galvanized using Zn–Ni (10 wt.%) alloy, were dipped into the zirconium precursor solution. The samples were dried up at room temperature and subsequently heated at 100 °C for 30 min. The deposition and drying processes of ZrO_2_ coating were repeated 3 times. The final thermal treatment was at 380 °C for 1 h.

The System ZC was prepared using similar procedures: firstly, the alloy coating Zn–Co (3 wt.%) was electrodeposited from an electrolytic solution, which contains ZnSO_4_·7H_2_O:CoSO_4_·7H_2_O:NH_4_Cl:H_3_BO_3_—100:120:30:25 g/L (Valerus Ltd., Sofia, Bulgaria). The pH value of the electrolyte was ~ 4.0, and the cathodic current density was 2 A/dm^2^. In addition, soluble zinc anodes (Valerus, Sofia, Bulgaria) and additives ZC-1 (wetting agent—20 mL/L, IPC-BAS, Sofia, Bulgaria) and ZC-2 (brightener—2 mL/L, IPC-BAS, Sofia, Bulgaria) were used [16]. The low-carbon steel samples, previously galvanized using Zn–Co alloy, were immersed into the zirconium precursor solution, following the abovementioned dip coating and treatment procedures.

For comparison, an ordinary zinc coating was obtained from solution having a composition of 150 g/L ZnSO_4_·7H_2_O, 30 g/L NH_4_Cl and 30 g/L H_3_BO_3_ under the following conditions: pH 4.5–5.0, cathodic current density 2 A/dm^2^, soluble zinc anodes and 2 additives: wetting agent (AZ1—40 mL/L, IPC-BAS, Sofia, Bulgaria) and brightener (AZ2—10 mL/L, IPC-BAS, Sofia, Bulgaria) [16]. The thickness of all investigated samples was about 11–12 µm.

### 2.2. Investigations with SEM/EDX Methods

The SEM/EDX investigations were carried out via scanning electron microscopy unit ZEISS Evo 10 (Oxford Instruments, Oxford, UK) in a high vacuum mode using secondary electron imaging and 25 keV accelerating voltage. The elemental composition of the samples was studied with energy dispersive spectroscopy (EDS) probe Oxford Ultim Max 40 and the results were compiled using the AZtec Live—Advanced software, version 6.1. The EDX analyses of the samples were realized in four different points.

### 2.3. AFM Studies and Hydrophobicity Measurements

Atomic Force microscope NanoScopeV system with Nanoscope software, Bruker Ltd., Mannheim, Germany, (operating in a tapping mode in air at room temperature, Cantilever force—40 N/m; resonance frequency of 300 kHz) was applied. The scanning rate was 1 Hz. The roughness analysis enables us to obtain the parameter Ra, the latter being an arithmetic average of the absolute values of the surface height deviations, obtained based on the mean plane, while Rq is the root-mean-squared average of height deviations, received from the mean image data. The water contact angles (WCAs) were evaluated with Ramé-Hart automated goniometer model 290 with DROP image advanced v2.4 (Succasunna, NJ, USA) at ambient temperature. Water drops having a volume within the 2–5 μL range were created and deposited with Ramé-Hart automatic dispensing system. The contact angles were measured from 10 consecutive drops positioned at random sample locations. The values of the mean angle and the mean error were obtained based on them. The contact angle values allow us to determine the wettability of the investigated surfaces [19]. 

### 2.4. Chemical and Phase Compositions 

The X-ray photoelectron spectroscopy (XPS) was used for the identification of the chemical composition and electronic structure of the films. The measurements were carried out on AXIS Supra electron-spectrometer (Kratos Analitycal Ltd., Manchester, UK) by the application of achromatic AlKα (photon energy of 1486.6 eV) having charge neutralization system. The binding energies (BEs) were calculated with an accuracy of ±0.1 eV, using the C1s line at 284.6 eV (due to adsorbed hydrocarbons). The areas and binding energies of C1s, O1s, Zn2p and Zr3d photoelectron peaks were monitored to evaluate the chemical composition of the films. Using the commercial data processing software ESCApe^TM^, version 1.2.0.1325 of Kratos Analytical Ltd., Manchester, UK, the concentrations of the different chemical elements (in atomic %) were rated. The sample surfaces were studied after etching. It was performed on VG ESCALAB II electron spectrometer with Ar+ ions with 3 keV of energy, a current density of 10 µA/cm^2^ and an etching rate of 2 nm/min.

### 2.5. XRD Analyses

The X-ray diffraction analysis allows us to define the phase composition of the samples by using an X-ray diffractometer (CuKα radiation; generator voltage 40 kV), equipped with a PW1830 generator and a PW1050 goniometer manufactured by Philips. Data were obtained within the angular range of 5–90° 2-theta with a step of 0.05° 2-theta and exposure of 3 s. The phase analysis was performed using the HighScore Plus 3.0 program, Inorganic Crystal Structure Database (ICSD) and Powder Diffraction File™ (PDF-2 2023).

### 2.6. Electrochemical Tests

Classical widespread electrochemical methods have been used with the aim to determine the anticorrosion properties of the sample systems and those of ordinary zinc. These are the potentiodynamic (PDP) polarization method (anodic and cathodic curves, potential range varying between −1.2 V and 0 V relative to SCE, scan rate 1 mV/s) as well as “open-circuit potential (OCP)”and polarization resistance (Rp) measurements. The latter was realized by applying a “Corrovit” unit (“Tacussel”, Villeurbanne, France). These methods and their peculiarities were applied by the authors previously for other test metallic/alloy objects [16]. The investigations were carried out after 15 min stabilization of the corrosion potential in a three-electrode experimental cell having a volume of 300 mL. Saturated calomel electrode was the reference electrode and platinum wire was the counter electrode. Potentiostat Model VersaStat 4 PAR, AMETEK, Oak Ridge, TN, USA was applied to realize the PDP and OCP investigations.

### 2.7. Data Reproducibility and Corrosive Medium

Five specimens of every sample type were studied electrochemically in a model corrosive test medium (5% NaCl solution).

## 3. Results

### 3.1. SEM and EDX Measurements

**Figure 1:**
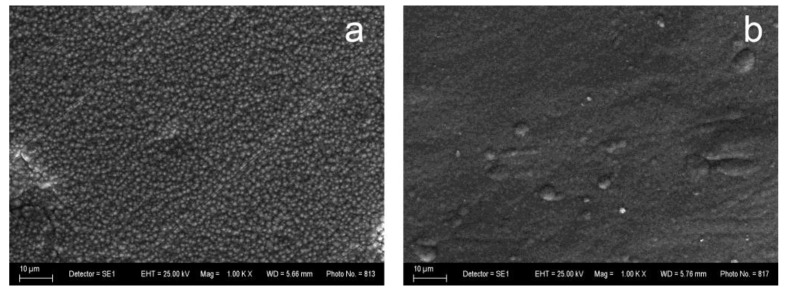
SEM analyses of the (**a**) Zn–Ni and (**b**) Zn–Co sub-layers (magnification 1000).

**Figure 2:**
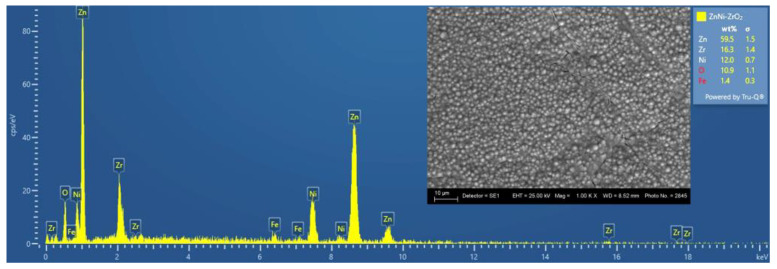
EDX/SEM analyses of the System ZN (magnification 1000).

**Figure 3:**
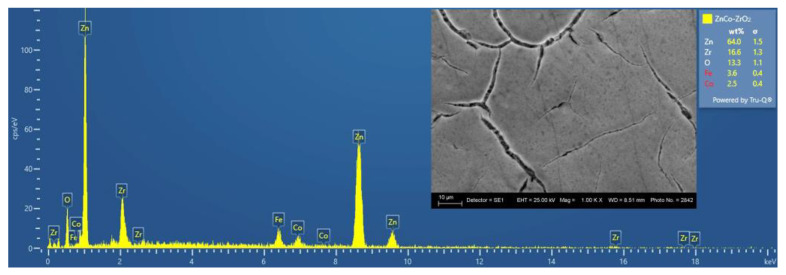
EDX/SEM analyses of the System ZC (magnification 1000).

The SEM photograph of Zn–Ni alloy coating reveals homogeneous fine-grained surface morphology, while the Zn–Co alloy possesses a smooth surface, covered with some micro-crack zones of different shapes and widths (Figure 1). The EDX analyses were performed on the previously etched samples (the procedure is described in Section 2.4). Figure 2 and Figure 3 reveal that the surfaces of the ZN and ZC systems repeat in detail the morphological features of the corresponding sub-layer. The results, obtained via SEM analyses, clearly show that the nature of the zinc alloy sub-layer significantly influences the morphology of ZrO_2_ top-layer, which is a result most probably arising from the different thermal expansion coefficients and the surface roughness of Fe, the Zn–Co and Zn–Ni alloys, as well as that of ZrO_2_.

### 3.2. AFM Analyses and Surface Hydrophobicity

**Figure 4:**
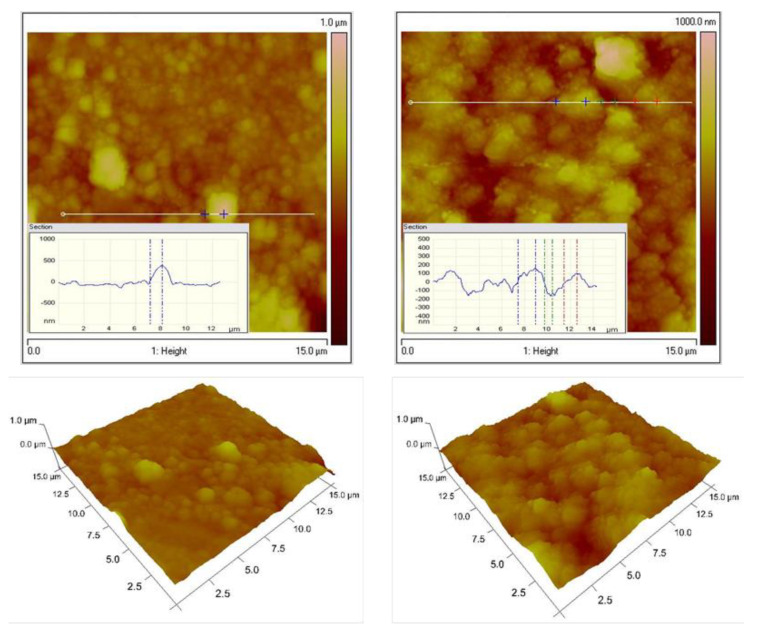
AFM 2D and 3D images of LCS (**left**); LCS/Zn (**right**).

**Figure 5:**
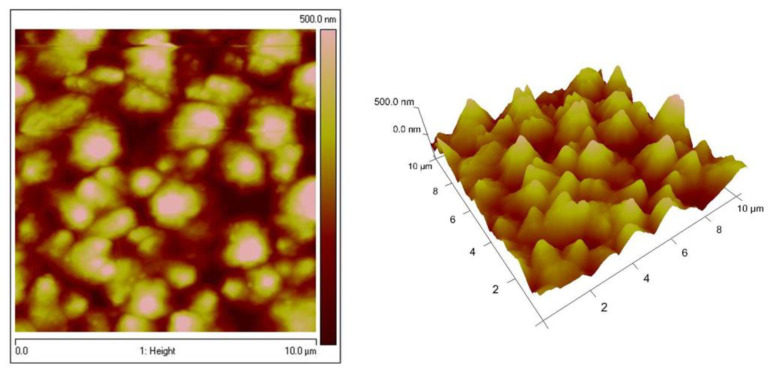
AFM (2D and 3D) images of Zn–Ni substrate.

**Figure 6:**
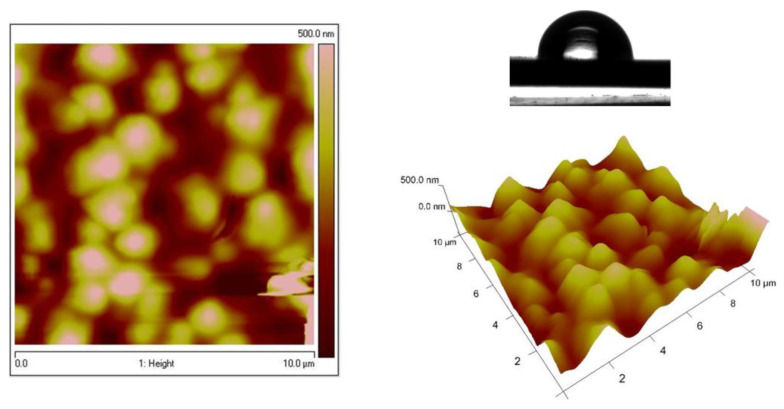
AFM images (2D and 3D) of System ZN and hydrophobicity of the surface.

Figure 4 represents the topography of the low-carbon steel (LCS) and galvanized steel (LCS/Zn) substrates for comparison to those of the ZN and ZC systems. The roughness values and corresponding water contact angles are shown in Table 1. The AFM images of Zn–Ni galvanized steel reveal a rough and grained surface, similar to those of steel and galvanized steel substrates (Figure 5). Figure 6 shows the dense topography without any visible pores, cracks, etc., of the System ZN after thermal treatment. The coating also has no visible detached parts from the substrate, which proves its good adhesion. The average roughness Ra is 105 nm, while Rq is 131 nm.

**Figure 7:**
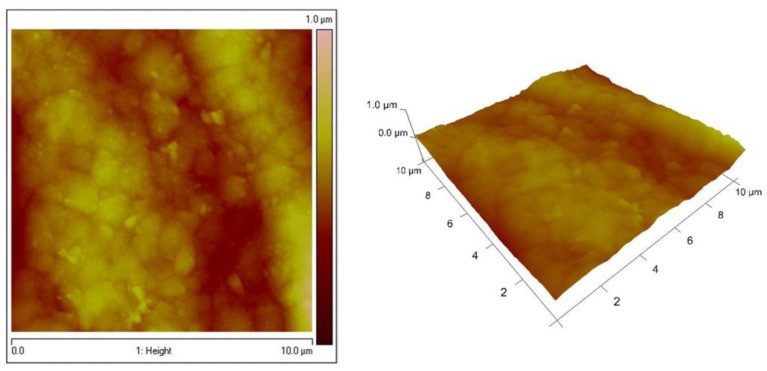
AFM images (2D and 3D) of Zn–Co substrate.

**Figure 8:**
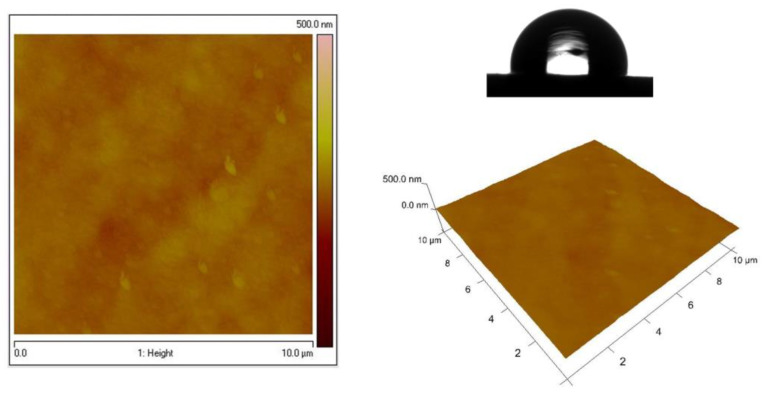
AFM images (2D and 3D) of System ZC and hydrophobicity of the surface.

The surfaces of Zn–Co substrate and the System ZC shows significantly smoother surface, which are demonstrated in Figure 7 and Figure 8, respectively. These data confirm the conclusions, based on the results of the SEM analyses, that the type of the previously electrodeposited zinc alloy coating is responsible for the final morphology of the top ZrO_2_ film. Table 1 represents the WCAs for System ZN, System ZC and different types of low carbon steel substrates.

The low-carbon steel substrate shows a typical hydrophobic surface character having a WCA value of 92°, while the steel, coated with electrodeposited Zn coating, has a much higher WCA value of 122 ± 2°. The modification of Zn with Ni or Co leads to a significantly decreased hydrophobicity. The deposition of ZrO_2_ coating upon the Zn alloys and subsequent heat treatment maintains the hydrophobic nature of the surface of the final ZN and ZC systems. The WCA values of the ZN and ZC systems are close to those of the corresponding sub-layers. 

### 3.3. XRD Analyses

**Figure 9:**
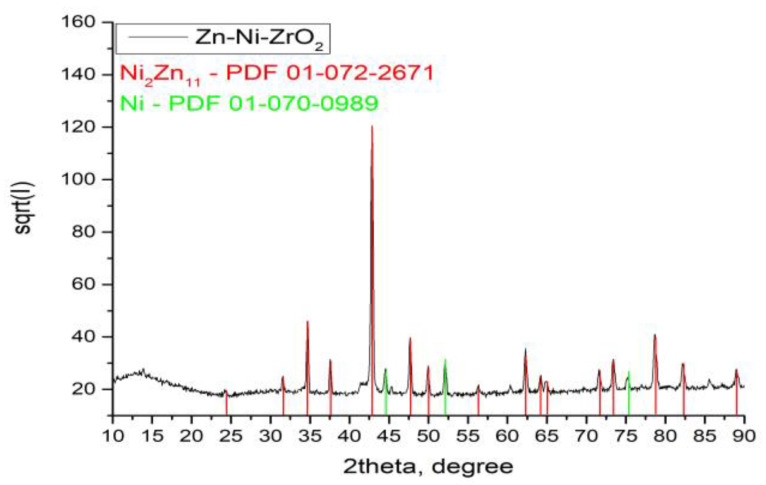
XRD of System ZN.

**Figure 10:**
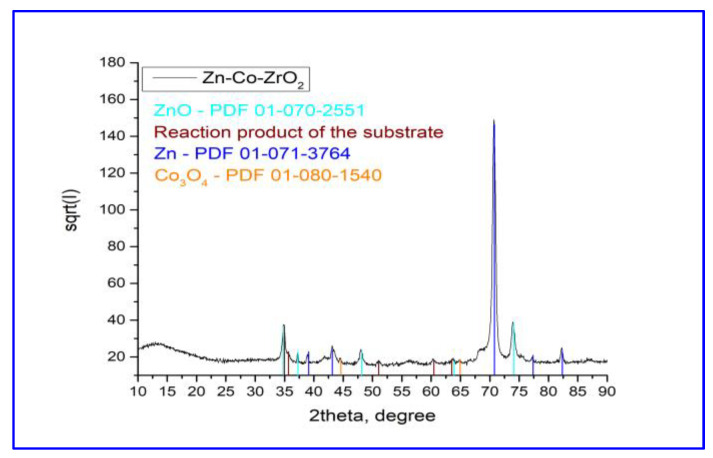
XRD of System ZC.

The System ZN containing nickel has a significantly simple phase composition (Figure 9). The co-deposition of nickel and zinc and subsequent annealing results in the formation of an intermetallic compound of composition Ni_2_Zn_11_. Any peaks, belonging to the ZrO_2_ crystallographic phases, have not been identified, most probably due to the amorphous nature of the top inorganic coating. A uniform coating was formed on the surface of the System ZC, in which the diffraction peaks of the metallic zinc were wider than those normally obtained (Figure 10). This is a sign that most likely some of the cobalt was included in its lattice. On the other hand, the coating has a complex phase composition, which is associated with a partial oxidation of the surface. This leads to some additional formation of zinc oxide (ZnO) and cobalt oxide (Co_3_O_4_), which possess a low degree of crystallization. Some weakly expressed peaks between 2-theta 35 and 65° probably belong to a non-identified phase as a result of the interaction between the zirconia layer and the substrate constituents.

### 3.4. XPS Analyses

**Figure 11:**
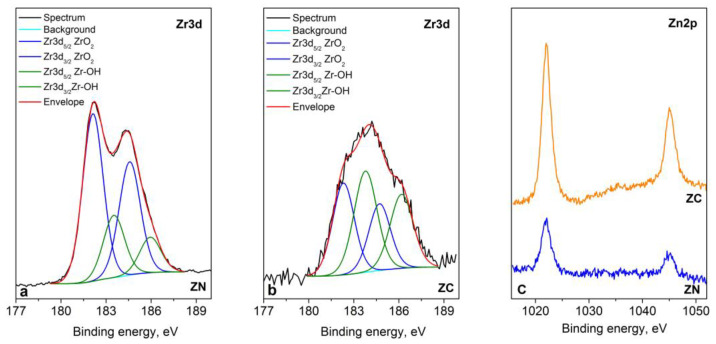
Deconvolution of Zr3d (**a**) ZN sample and (**b**) ZC sample and (**c**) Zn2p core level spectra of the systems.

**Figure 12:**
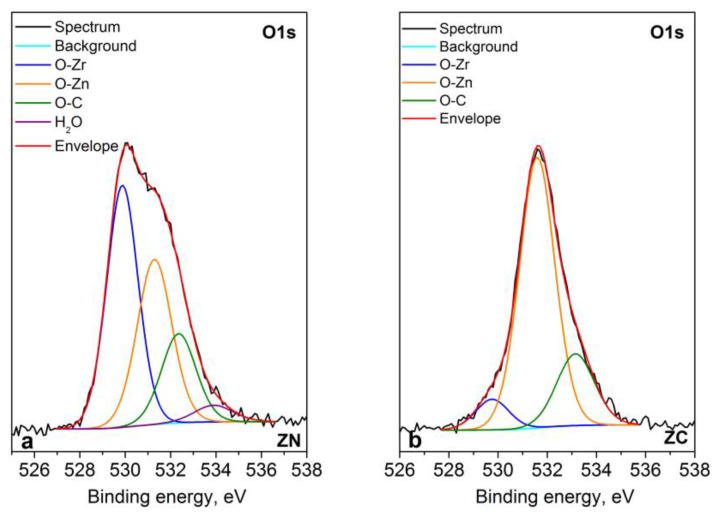
Deconvolution of O1s core level spectra of (**a**) ZN and (**b**) ZC systems.

The XPS analysis represents that the chemical elements Zn, Zr, C, O and Na are registered on the surface of the layers. The Zr3d peaks (presented in Figure 11a,b) are intensive with spin–orbit splitting between Zr3d_5/2_ and Zr3d_3/2_ from 2.4 eV and this shows that zirconia exists in the form of two phases: ZrO_2_ (182.2 eV) and ZrOH (183.6 eV) [20]. The shape of spectra and the binding energy of zinc at 1021.9 eV strongly suggest a 2^+^ oxidation state (Figure 11c). For a more precise determination of the state of zinc, the modified Auger parameter was also calculated, which is 2009.2 eV, and it corresponds to ZnО. The oxygen spectra are broad and asymmetric. A Lorentzian–Gaussian curve fitting into several components was applied in the oxygen spectra (Figure 12). The binding energy at ~529.8 eV corresponds to the O–Zr bond, while that at ~531.4 eV is attributed to O in the Zn crystal lattice. The last two components refer to the oxygen–carbon bond at ~532.7 eV and to the oxygen in the water molecule at ~534.0 eV (only for the ZN sample). The carbon spectra indicate that there is a layer of adventitious carbon contamination on the surface of the samples. Most samples, exposed to the atmosphere, have adventitious carbon on the surface. This statement is also proven by the components of the decomposed spectra of carbon, which are attributed to C–C, C–O–C and O–C=O bonds. Small amounts of sodium (0.7 at %) for ZN sample were registered, probably due to contamination.

### 3.5. Electrochemical Tests

#### 3.5.1. Measurement of the Polarization Resistance

**Figure 13:**
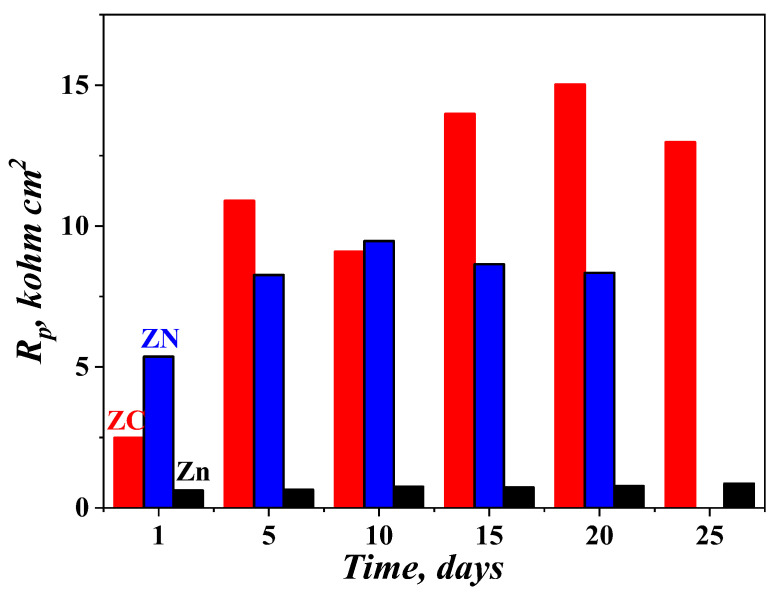
Polarization resistance of the investigated samples in 5% NaCl solution.

The polarization resistance in 5% NaCl solution, measured during a time period of 25 days for the ZC (Zn–Co/ZrO_2_), ZN (Zn–Ni/ZrO_2_) and the ordinary zinc coating systems can be observed in Figure 13. According to the figure, the *Rp* values of the ordinary zinc are the lowest ones (~800–900 ohms.cm^2^) and they are very close in value. System ZC demonstrates an almost increasing tendency (except for the 10th day) and the highest peak of *Rp* values on the 20th day (~15,000 ohms.cm^2^). This system reaches the polarization resistance value of about 13,000 ohms.cm^2^ at the end of the test. System ZN represents the highest *Rp* value at the 10th day (~9500 ohms.cm^2^), which is very close to the value of the same parameter of System ZC. The *Rp* values of System ZN decrease until the 20th day, after which some separate “red points” appear on its surface, indicating the presence of the so-called “red rust”, i.e., the corrosion process has reached the low-carbon steel substrate and the latter has begun to dissolve as a result of the corrosion attack. Bearing in mind this behavior, the *Rp* measurement of the system is stopped. Contrary to this, both System ZC and the ordinary zinc do not show any “red rust” zones, which is a sign of their better protective characteristics during the test period in a corrosive environment. 

#### 3.5.2. Potentiodynamic Polarization Curves of Fresh Samples

**Figure 14:**
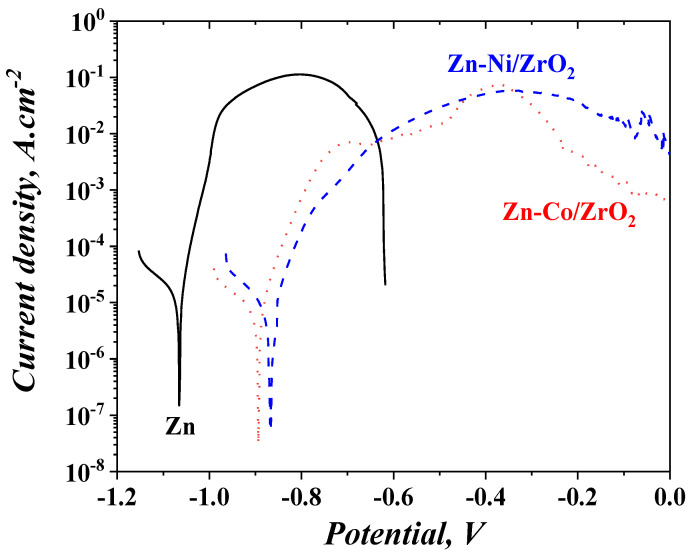
Potentiodynamic polarization curves of the fresh samples in 5% NaCl solution (vs. SCE).

The PDP curves of the investigated usual zinc coating and both systems are shown in Figure 14. It can be observed that the corrosion potential of the ordinary zinc coating is manifested at a more negative value, compared to that of System ZC and System ZN. In addition, Zn represents a curve, which is very short in the anodic branch, compared to both systems. This observation means that this metal lasts a shorter time period at external anodic polarization in that medium. The anodic curves of System ZC and System ZN are very similar in their course, although some differences appear—System ZC shows a zone with lower current density values, for example, in the potential interval between −0.7 and 0.4 V. It can also be registered that the anodic part of the PDP curve of System ZN seems to be longer than that of System ZC. However, the anodic curve of System ZN is positioned at higher current densities after the potentials of −0.3 V, i.e., the anodic process is realized more intensively.

Table 2 presents the most important parameters of the PDP curves: corrosion potential E_corr_ and corrosion current density I_corr_. The results indicate that the zinc sample has the most negative potential value compared to both other systems, which means that the latter are more noble. This can be expected when keeping in mind the nature of the alloying elements and the final surface oxide layer. It can also be concluded that the corrosion current densities of the systems are much lower compared to that of zinc, i.e., their corrosion resistance and protective ability are greater.

#### 3.5.3. Long Period Test (25 Days in Test Medium) of the Samples Presented by Potentiodynamic Polarization Curves 

**Figure 15:**
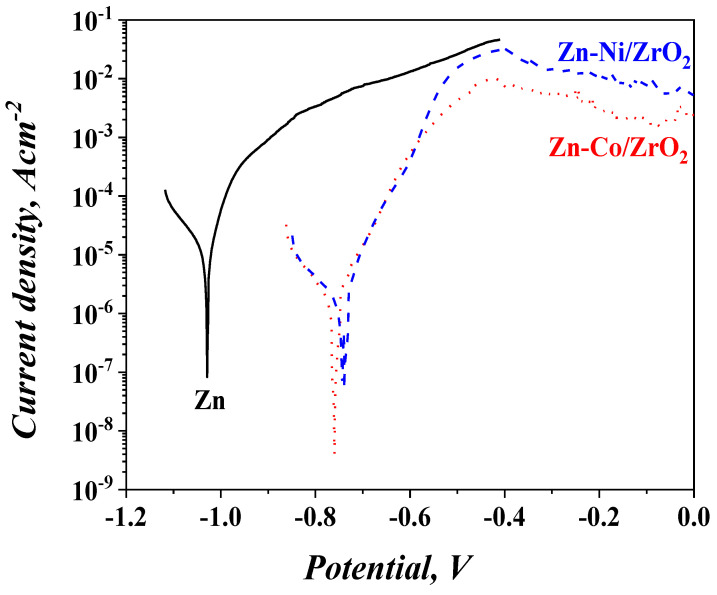
Potentiodynamic polarization curves of the investigated samples after 25 days of continuous immersion in 5% NaCl solution (vs. SCE).

**Figure 16:**
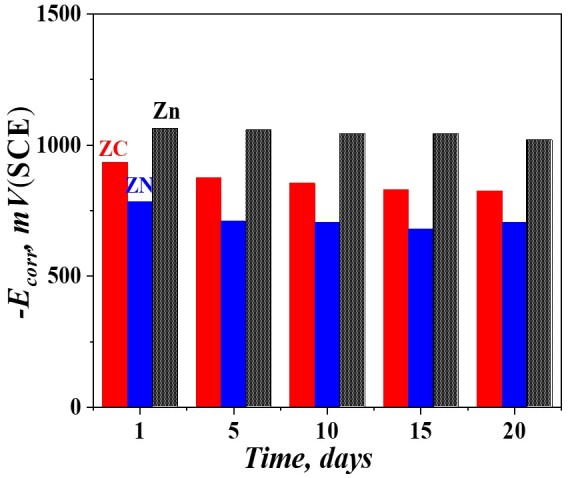
Open-circuit potential (OCP) values of the investigated samples after 25 days of immersion in 5% NaCl solution (vs. SCE).

The corrosion characterization of the samples has also been carried out through the application of curves after the 25-day immersion in the corrosion medium (Figure 15 and Table 3). The corrosion potential (E_corr_) of both systems ZC and ZN are additionally strongly shifted in a positive direction with about 140 mV, i.e., they become more noble. E_corr_ of the pure zinc is also positively shifted with approximately 40 mV (compare Figure 14 and Figure 16). The reason for this result is most probably the changes in sample surface as a result of the corrosive treatment. As it is well known, the corrosion potential depends on the surface oxidation state and on the appearance of different corrosion products, and it can be expected that some differences will appear in its value. Compared to the results from Table 2, it can be concluded that the I_corr_ values of ZN and ZC systems become about four times lower, while that of the ordinary zinc remains the same. In the case of the ordinary zinc, the presence of some newly appeared corrosive products might affect the potential shift. When both systems are under corrosion attack, some other corrosion products are obtained on the surface, influencing the corrosion parameters. Another important reason could be the presence of cracks, pores or some surface non-homogeneities, which could ensure a faster access of the corrosive medium deep inside.

The results proved that the influence of the surface oxide layer and of the subsequently newly appeared corrosion products positively affect the corrosion behavior and protective ability of the investigated systems in that test medium.

#### 3.5.4. Open-Circuit Potential (OCP)

The results for the OCP values of the tested objects are shown in Figure 16. It is well visible that the values of the ordinary zinc are the most negative ones in comparison to those of ZC and ZN systems, which means that this coating plays a sacrificial role and will dissolve earlier, ensuring an appearance of zinc-based corrosion products. Contrary to this, the OCP values of both systems are more positive (noble), most probably due to the appearance of protective barrier layers of corrosive products. It could be supposed that the appearance of “red rust” for System ZN results from the presence of cracks and surface defects on the layer, leading to a faster corrosion attack deep inside. 

## 4. Discussion

The electrochemical data, obtained during the experiments conducted with the application of both zinc-based alloys as sub-layers, show enhanced anticorrosion characteristics of the systems. For example, ZN and ZC demonstrate a positive potential shift of the corrosion potential (E_corr_) compared to that of the usual zinc, which is proof for their better-expressed “nobility”. This potential shift is observed both for the fresh samples and for the samples after prolonged treatment in the corrosion medium.

A similar tendency can be observed in view of the “open-circuit potential” measurements—the potential value of the zinc is more negative, compared to both systems during the whole investigation period of time. In addition, the corrosion current density values of ZN and ZC taken from the PDP curves are lower compared to that of the pure zinc. 

Potentiodynamic curves of ZN and ZC are about 400 mV longer than that of the zinc, i.e., these systems last longer under conditions of external anodic polarization.

The polarization resistance measurements during a 25-day time period confirm in general the results from the accelerated tests. However, bearing in mind the obtained results, System ZN seems to be insufficiently resistant. One possible reason could be the presence of more cracks and pores, as well as a greater surface inhomogeneity, which makes the penetration of the chloride ions deep inside easier, leading to the appearance of some “red rust” zones and the deterioration of the protective characteristics.

Compared to the other investigations on this topic, which are scarce, the following conclusions could be summarized: (i) the other authors use spray pyrolysis to obtain the ZrO_2_ layers onto aluminized steel with different content; and (ii) the surface morphology, for example, is different due to the characteristics of the applied method. Bearing all these peculiarities in mind, it seems to be very difficult to compare the results obtained for all systems. However, based on the experimental results, it can be concluded that System ZC demonstrates very good protective characteristics and it can be successfully applied for the protection of low-carbon steel in chloride-containing media.

It is known that several physical–chemical parameters can affect the anti-corrosion behavior of the barrier oxide coatings: surface morphology, hydrophobicity, the presence of specific crystalline phases and/or the presence of amorphous phase, the presence of defects, pores, etc.

The surface morphology of the zirconia layers in our case is influenced by the type of Zn contained in the sub-layer: the modification with Co of galvanized steel leads to the formation of smooth films. On the other hand, the presence of nickel-doped galvanized steel promotes the formation of a finely grained ZrO_2_ surface. These results have also been proven by the estimated values of the average surface roughness (105 nm vs. 75 nm for ZN and ZC, respectively). Keeping in mind the obtained experimental results, it seems that the surface characteristics of the corrosion-resistant coatings do not have a well-expressed relationship with the corrosion stability of the investigated systems.

The phase composition and/or the presence of an amorphous phase of ZrO_2_ coating is another important factor, influencing its corrosive protection properties. The XRD analyses have not registered any crystallographic phases of zirconia, so the ZrO_2_ films obtained in both systems possess an amorphous nature. These findings were proven through the studies of other researchers. According to the results obtained in [21,22], the amorphous phase leads to a lower conductivity (ionic and electronic), thus retarding the electrochemical reactions on the surface. It has been proven that the good anti-corrosion properties of the ZrO_2_ films are due to their high density and partially amorphous character [23]. In the case of ZC system, the appearance of new phases, consisting of low crystalline ZnO and Co_3_O_4_ oxides, can additionally improve the protective characteristics of the system in comparison to the System ZN. It could be assumed that the presence of these oxides can impede the penetration of the corrosion agents, thus slowing down the rate of the corrosion process due to their barrier properties.

Further, the surface hydrophobicity is also responsible for the high anti-corrosion efficiency of the systems. As a rule, it inhibits the corrosion process by limiting the access of surface corrosive species (water, halide ions, etc.) and interaction. Subsequently, the destruction of the metal oxide films becomes slower. Jothi et al. have proven that hydrophobicity (122°) of the surface improves the corrosion resistance. They have obtained polyurethane-Pd-ZrO_2_ nanocomposites (in different combinations), possessing good anti-corrosion performance in a 3.5% NaCl medium [24]. On the other hand, our research group has revealed the absence of a direct correlation between the high contact angle of water and better anti-corrosion protection [25]. Huang and co-authors [26] also found that superhydrophobic TiO_2_ nanotubes possess low corrosion resistance. 

In our case, the lower contact angle of the ZN and ZC systems, compared to that of Zn-coated low-carbon steel, does not lead to the deterioration of the corrosion characteristics. It can be assumed that this is due to the presence of micro- or nano-pores, which are connected in subchannels. These subchannels enable the penetration of the aggressive agents (Cl anions) through the ZrO_2_ coating. The zirconia layer has a densely amorphous structure, suggesting that some of these channels are also narrower than those of galvanized steel. This induces a limitation of the areas, subjected to a corrosive medium. Similar suppositions have been suggested by Yu et al., which can explain the anti-corrosion properties of a three-layer composite coating (containing TiO_2_ and ZnO layers) [27]. It could be summarized that the synergism between the abovementioned physicochemical features of the surface ensures the good corrosion-protective properties of the systems presented in this article.

## 5. Conclusions

The results presented in the article have established that the newly obtained systems based on low-carbon steel covered with Zn–Co or Zn–Ni alloy (as sub-layer) and an amorphous hydrophobic ZrO_2_ sol–gel layer (as the top layer) showed generally improved protective characteristics, compared to those of the ordinary zinc in a model medium with chlorine ions. The System ZC possesses a lower corrosion current density value (1.6.10^−6^ A.cm^−2^) and improved polarization resistance (13,000 ohms.cm^2^) to those of ordinary zinc and System ZN, which is due to the formation of poorly crystallized oxides (ZnO and Co_3_O_4_, both of which play a role as a barrier toward corrosive agents).

The polarization resistance of both systems at the end of the 25-day prolonged test time period is much higher, compared to that of the usual zinc; however, some “red rust” zones appear on ZN (most probably due to the presence of cracks), which is a sign of lower protective characteristics. Contrary to this, System ZC reveals great potential for industrial applications, owing to both the good protective characteristics and the advantages of the sol–gel method: good adhesion to the metal substrates and a possibility to deposit layers on large surface areas with unique shape.

## Figures and Tables

**Table 1:** Water contact angles for System ZN, System ZC and different types of low-carbon steel substrates (LCSs).

Sample	LCS	LCS /Zn	LCS /Zn–Ni	LCS /Zn–Co	LCS/Zn–Ni- ZrO_2_ (ZN)	LCS/Zn–Co- ZrO_2_ (ZC)
WCA (°)	92	122	97	95	93	90
Rp (nm)	59	69	68	117	131	9.5
Ra (nm)	46	56	53	92	105	7.4

**Table 2:** Electrochemical parameters of the PDP curves from Figure 14.

Sample	E_corr_, V	I_corr_, A.cm^−2^
Zn	−1.065	1.8 × 10^−5^
Zn–Co/ZrO_2_	−0.895	6.5 × 10^−6^
Zn–Ni/ZrO_2_	−0.868	7.6 × 10^−6^

**Table 3:** Electrochemical parameters of the PDP curves from Figure 15.

Sample	E_corr_, V	I_corr_, A.cm^−2^
Zn	−1.029	1.8 × 10^−5^
Zn–Co/ZrO_2_	−0.763	1.6 × 10^−6^
Zn–Ni/ZrO_2_	−0.748	1.7 × 10^−6^

## Data Availability

Data are contained within the article.
